# Staphylococcal Scalded Skin Syndrome in a Tertiary Pediatric Hospital over an 11-Year Period

**DOI:** 10.3390/children13070965

**Published:** 2026-07-21

**Authors:** Magdalini Louka, Maria Lekaditi, Manthoula Valari, Filippos Filippatos, Athanasios Michos

**Affiliations:** 1 Infectious Diseases and Chemotherapy Research Laboratory, First Department of Pediatrics, Medical School, “Aghia Sophia” Children’s Hospital, National and Kapodistrian University of Athens, 11527 Athens, Greece; mlekaditi@hotmail.com (M.L.); filippat@med.uoa.gr (F.F.); amichos@med.uoa.gr (A.M.); 2 Pediatric Dermatology Unit, First Department of Pediatrics, Medical School, “Aghia Sophia” Children’s Hospital, National and Kapodistrian University of Athens, 11527 Athens, Greece; mvalari@yahoo.com

**Keywords:** staphylococcal scalded skin syndrome, *Staphylococcus aureus*, antibiotic therapy, antimicrobial resistance, skin infections

## Abstract

**Highlights:**

**What are the main findings?**

- In this 11-year retrospective study conducted at a tertiary pediatric hospital, the incidence of staphylococcal scalded skin syndrome increased after 2017 and peaked in 2019, with half of all cases occurring during 2018–2019.
- Oxacillin resistance remained low and stable, whereas mupirocin resistance increased. Exploratory regression analyses showed that child nasal *Staphylococcus aureus* carriage was associated with microbiological confirmation and non-penicillin antimicrobial resistance.

**What are the implications of the main findings?**

- Staphylococcal scalded skin syndrome management in children should be guided by local microbiological surveillance and regional antimicrobial resistance patterns, especially where clindamycin or topical decolonization strategies are used.
- Rising mupirocin resistance, together with the association between child nasal *Staphylococcus aureus* carriage and clinically relevant non-penicillin antibiotic resistance, supports cautious topical antibiotic use, targeted decolonization, and consideration of household transmission.

**Abstract:**

**Background/Objectives:** Staphylococcal scalded skin syndrome (SSSS) is a toxin-mediated skin disorder caused by *Staphylococcus aureus* (*S. aureus*), mainly affecting young children. This study aimed to describe the epidemiology, clinical features, microbiology, and management of pediatric SSSS and to explore factors associated with culture positivity, antimicrobial resistance, and complications. **Methods:** Retrospective cohort study of children hospitalized with SSSS at the major tertiary pediatric hospital in Greece, from January 2009 to December 2019. Exploratory association testing and parsimonious logistic regression were performed. **Results:** Overall, 51 children with a mean (±SD) age 2.5 ± 2 years were identified. Prior to diagnosis, 70.6% (36/51) of children had not received any antibiotic treatment. *S. aureus* was isolated from cultures in 43.1% (22/51) of cases. Carriage of *S. aureus* was detected in 34.0% (16/47) of children and 37.0% (10/27) of parents. Antibiotic resistance (%) for *S. aureus* was penicillin 95.5%, oxacillin 4.5%, erythromycin 4.5%, clindamycin 9.1%, mupirocin 40.9%, and fusidic acid 18.2%. Annual incidence of SSSS was estimated at 0.00–0.32/1000 hospitalizations from 2009 to 2016, but increased after 2017, reaching the highest incidence in 2019 (1.05/1000 hospitalizations); 50.7% of cases occurred in 2018–2019 (*p* < 0.001). During this period, resistance to oxacillin (MRSA strains) did not change (*p* = 0.53), whereas resistance to mupirocin increased over time, from 0% in 2009–2014 to 34.6% in 2018–2019 (*p* = 0.05). Penicillin was excluded from the composite drug-resistance outcome. Child nasal *S. aureus* carriage was independently associated with positive *S. aureus* culture (adjusted odds ratio [aOR] 4.18, 95% CI 1.14–15.27; *p* = 0.031) and with non-penicillin antibiotic resistance (aOR 25.68, 95% CI 2.64–249.50; *p* = 0.005). Elevated inflammatory markers were independently associated with complications (aOR 13.66, 95% CI 1.47–126.64; *p* = 0.021). Regarding treatment, clindamycin was the first-line agent in 58.8% (30/51) and was used as monotherapy in 49.0% (25/51). **Conclusions:** A notable increase in the incidence of SSSS was detected after 2017. Oxacillin resistance remained stable, but mupirocin resistance increased. Child nasal *S. aureus* carriage was associated with culture positivity and clinically relevant non-penicillin antibiotic resistance. Continuous surveillance is necessary as regional resistance patterns should guide therapy.

## 1. Introduction

Staphylococcal Scalded Skin Syndrome (SSSS) is a blistering dermatosis caused by exfoliative toxins produced by certain *Staphylococcus aureus* (*S. aureus*) strains [1]. Only about 5% of isolates produce causative toxins, primarily exfoliative toxin A and exfoliative toxin B [2]. These toxins disseminate hematogenously and target desmoglein-1, disrupting epidermal cohesion and leading to skin exfoliation [2].

SSSS primarily affects children under six years of age, including neonates [1,3]. It presents with painful macular erythema and early desquamation, predominantly affecting the head, neck, and flexural areas (axillae, inguinal folds, and gluteal cleft), with generalized erythema typically developing within 48 h. As the disease progresses, flaccid bullae and superficial erosions appear in areas of friction, followed by widespread desquamation, while characteristic perioral and periorbital crusting with fissuring (“sad face”) is often observed [3]. Common associated findings include fever, irritability, and poor feeding [1,3]. Diagnosis is primarily clinical, based on generalized erythroderma, a positive Nikolsky sign, and the absence of mucosal involvement [1,3]. Lesions are mostly sterile; therefore, cultures should be obtained from potential infection foci, including the nasopharynx, conjunctiva, perioral and perianal areas, and in neonates, the umbilical stump [1]. Blood cultures are usually negative [3]. Biopsies are rarely required and show epidermal detachment at the granular layer, distinguishing SSSS from pemphigus or Stevens–Johnson syndrome [1,3].

SSSS is a dermatologic emergency requiring hospitalization, intravenous antibiotic therapy, and supportive measures [4]. Empirical treatment includes penicillinase-resistant penicillins, first-generation cephalosporins, or clindamycin, while glycopeptides are reserved for resistant strains based on local antimicrobial susceptibility patterns [3,5,6]. Complications may involve secondary infections, sepsis, or electrolyte imbalances. Mortality in children is around 3%, but in adults it can exceed 50% [4].

The incidence of SSSS differs by region and study [1]. In the United States, the annual incidence is 7.67 per million children and 45 per million among those under two years of age [7]. European rates are lower (0.56 cases per million inhabitants annually in France) [8], while China reports 696.4 per million [6].

In Greece, data on pediatric SSSS remain limited [9]. This study aims to describe the epidemiological and microbiological characteristics of children diagnosed and hospitalized with SSSS at the major Greek tertiary Children’s Hospital over an 11-year period in the pre-COVID-19 era, to evaluate *S. aureus* antimicrobial resistance patterns, and to explore associations between carriage, culture positivity, resistance, treatment modality, and complications.

## 2. Materials and Methods

### 2.1. Study Design, Setting, and Reporting Framework

This retrospective, single-center observational cohort study was conducted at “Aghia Sophia” Children’s Hospital, the largest tertiary pediatric hospital in Greece, with 750 beds. The cohort included children aged ≤ 16 years who were hospitalized between 1 January 2009 and 31 December 2019. For each participant, the observation period extended from hospital admission to discharge; no post-discharge follow-up was undertaken. The study was reported according to the STROBE checklist for cohort studies (Supplementary File S1).

Hospital discharge records were screened using the International Classification of Diseases, 10th Revision (ICD-10) code L00 for a primary diagnosis of staphylococcal scalded skin syndrome (SSSS). Eligibility required hospitalization during the study period, age ≤ 16 years, and clinical documentation of generalized erythema with superficial blistering and/or exfoliation, a positive Nikolsky sign, and no mucosal involvement. These diagnostic criteria were used during the study period. The diagnosis was made by Pediatricians in the Emergency Department or inpatient Pediatric Department and confirmed by a pediatric dermatologist. Inclusion was not dependent on *S. aureus*, and children were eligible whether *S. aureus* was grown from cultures or not. All records that met these prespecified criteria were included in the final cohort.

### 2.2. Data Sources and Data Collection

Data were retrospectively abstracted from the medical records and entered into a pseudonymized, coded Microsoft Excel database. Collected variables included demographics, geographic origin, nationality, year and season of diagnosis, duration of hospitalization, initial diagnostic classification, pediatric dermatologist evaluation, clinical outcomes, underlying conditions, preceding infections, antibiotic exposure during the previous six months, laboratory findings, suspected entry sites, microbiological results, antimicrobial treatment, and complications. Annual numbers of all pediatric hospitalizations, used as denominators for incidence calculations, were obtained from hospital administrative records. For each child, parental nasal carriage was classified as positive when *S. aureus* was detected in at least one sampled parent.

### 2.3. Variables and Outcome Definitions

The principal descriptive outcomes were annual SSSS incidence, microbiological confirmation, antimicrobial resistance patterns, treatment, complications, and in-hospital outcome. Annual incidence was calculated as the number of SSSS admissions divided by the total number of pediatric hospitalizations in the same calendar year and multiplied by 1000. Microbiological confirmation was defined as growth of *S. aureus* from at least one culture obtained from a suspected infection focus; screening nasal cultures were analyzed separately as carriage. Child and parental nasal carriage, prior antibiotic use, the late study period (2018–2019), inflammatory marker category, and treatment modality were evaluated as potential predictors. The elevated inflammatory marker category used thresholds of white blood cell count > 15,000/μL and C-reactive protein ≥ 20 mg/L. Non-penicillin resistance was defined as resistance to at least one of oxacillin, erythromycin, clindamycin, mupirocin, or fusidic acid; penicillin was excluded because resistance was near universal and was not a clinically informative first-line anti-staphylococcal comparator. Complications were classified as minor (conjunctivitis, dehydration, or antibiotic-related adverse reactions) or major (hospital-acquired infection or intensive care unit admission); “any complication” included either category. For exploratory analyses, combination/advanced intravenous therapy comprised dual intravenous therapy, initial dual therapy followed by monotherapy, or treatment escalation or switching because of inadequate clinical response.

### 2.4. Microbiological Identification and Antimicrobial Susceptibility Testing

*S. aureus* cultures were obtained from potential infection sites, including perioral, perianal, conjunctival, and lesion-adjacent skin sites when present. Carriage was assessed exclusively using nasal swabs. Routine parental nasal screening was not performed and was requested at the discretion of the treating physicians, mainly in case of household transmission or decolonization. Blood cultures were obtained at admission and repeated only when clinically indicated, such as in the presence of fever or signs of systemic infection. All available isolates were analyzed. Only antibiotics routinely tested by the hospital microbiology laboratory during the study period were included in the analysis, as these represent the antimicrobial agents most commonly used for the management of pediatric *S. aureus* skin infections and SSSS. Susceptibility to penicillin, oxacillin, erythromycin, clindamycin, mupirocin, and fusidic acid was assessed using the Kirby–Bauer disk diffusion method in accordance with the applicable Clinical and Laboratory Standards Institute (CLSI) guidelines. Penicillin susceptibility was interpreted according to CLSI recommendations using both the inhibition-zone diameter and the appearance of the zone edge. Isolates with a zone diameter ≥ 26 mm and a fuzzy (“beach-like”) zone edge were considered penicillin susceptible, whereas isolates with a sharp zone edge were reported as penicillin resistant regardless of zone diameter. Mupirocin susceptibility was assessed using both 5-μg and 200-μg mupirocin disks. Isolates with inhibition zones around both disks were considered susceptible. Absence of an inhibition zone around the 5-μg disk with persistence of an inhibition zone around the 200-μg disk was interpreted as low-level mupirocin resistance, whereas absence of inhibition zones around both disks indicated high-level mupirocin resistance. All *S. aureus* isolates were screened for methicillin resistance using cefoxitin disk diffusion; cefoxitin-resistant isolates were further tested with the penicillin-binding protein 2a (PBP2a) Slidex methicillin-resistant *S. aureus* (MRSA) detection kit (bioMérieux, Marcy l’Étoile, France). The D-test was performed for erythromycin-resistant, clindamycin-susceptible isolates to detect inducible clindamycin resistance, and isolates with a positive D-test were classified as clindamycin resistant. Exfoliative toxin production was not tested.

### 2.5. Potential Sources of Bias and Study Size

Diagnostic misclassification was reduced by requiring characteristic clinical findings and confirmation by a pediatric dermatologist. To limit selection bias, all eligible hospitalizations during the prespecified 11-year period were included. Nevertheless, potential ascertainment bias remained because cultures and parental screening were obtained according to clinical judgment rather than a uniform study protocol. Therefore, tested denominators are reported explicitly, analyses involving carriage were restricted to participants with available testing, and parental carriage findings were interpreted as exploratory. The study size was determined by the total number of eligible SSSS admissions during the study period; no a priori sample size calculation was performed because the study constituted a census of a rare condition at the participating hospital.

### 2.6. Statistical Analysis

Normality of continuous variables was assessed using the Kolmogorov–Smirnov test. Continuous variables were summarized as mean ± standard deviation (SD) or median with interquartile range (IQR), as appropriate, and categorical variables as number and percentage. Age was analyzed continuously and in clinically relevant pediatric age groups. The inflammatory marker thresholds and calendar period groupings were prespecified as described above. Categorical variables were compared using the χ^2^ test or Fisher’s exact test, as appropriate, and non-normally distributed continuous variables were compared using the Mann–Whitney U test. All tests were two-sided, and *p* < 0.05 was considered statistically significant. Analyses were performed using IBM SPSS Statistics for Windows, Version 26.0. Annual SSSS incidence was expressed per 1000 hospitalizations. Temporal analyses compared the proportion of cases in 2018–2019 with that in 2009–2017 and evaluated resistance across four predefined periods (2009–2011, 2012–2014, 2015–2017, and 2018–2019).

Exploratory association testing and binary logistic regression were performed for two prespecified primary dependent variables: microbiological confirmation with *S. aureus* and clinically relevant non-penicillin antimicrobial resistance. Any complication was evaluated as a secondary dependent outcome. Candidate predictors included child and parental nasal carriage, the late study period (2018–2019), prior antibiotic use, the elevated inflammatory-marker category, *S. aureus* isolation, and treatment modality. Adjustment variables were selected a priori on the basis of clinical relevance and potential confounding. Because of the small cohort and sparse events, each model was intentionally parsimonious and included no more than two predictors. Child carriage was adjusted for the late study period in the culture-positivity model; parental carriage was adjusted for child carriage; child carriage and the late study period were mutually adjusted in the resistance model; and elevated inflammatory markers and combination/advanced intravenous therapy were mutually adjusted in the complications model. Results are reported as odds ratios (ORs) or adjusted odds ratios (aORs) with 95% confidence intervals (CIs). Missing data were not imputed; available case analyses were used, with denominators reported for each variable. Child nasal carriage data were available for 47 children and parental nasal carriage data for 27 families. In-hospital outcome data were available for all participants, and loss to follow-up was not applicable. No formal sensitivity analyses or adjustment for multiple comparisons were performed because of the exploratory nature and limited size of the study; consequently, the association analyses were interpreted cautiously.

## 3. Results

### 3.1. Participant Selection and Demographic Characteristics

The final cohort comprised 51 eligible children. Complete demographic and in-hospital outcome data were available for all participants. Child nasal carriage was assessed in 47/51 children, and parental nasal carriage was assessed in 27/51 families; analyses involving these variables used the corresponding available case denominators. Girls accounted for 51.0% (26/51), and the mean (±SD) age was 2.5 ± 2.0 years (Table 1).

### 3.2. Annual and Seasonal Distribution

**Figure 1:**
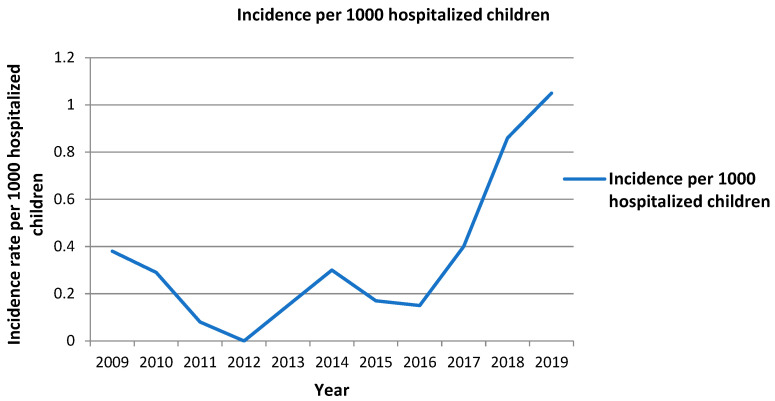
Annual incidence of staphylococcal scalded skin syndrome per 1000 hospitalized children in a tertiary pediatric hospital between 1/2009 and 12/2019.

Annual incidence ranged from 0.00 to 0.32 cases per 1000 hospitalizations from 2009 to 2016 but increased after 2017, reaching 0.85 in 2018 and 1.05 in 2019 (Figure 1). Over half the cases (50.7%, 26/51) occurred in 2018–2019 (*p* < 0.001). Seasonal variation was not significant (*p* = 0.340), though 31.4% (16/51) were reported during summer or autumn.

### 3.3. Clinical Presentation and Comorbidities

Underlying conditions were present in 15.7% (8/51): atopic dermatitis (*n* = 6), ichthyosis (*n* = 1), and nephrotic syndrome (*n* = 1). Before admission, 70.6% (36/51) had not received antibiotics, 27.4% (14/51) had received β-lactams, and 2.0% (1/51) had received topical antibiotics. At presentation, 76.5% (39/51) were correctly diagnosed with SSSS; the remainder were initially misdiagnosed with urticaria (13.7%, 7/51) or Stevens–Johnson syndrome (2.0%, 1/51).

Preceding upper respiratory tract infections occurred in 54.9% (28/51). Localized infections were identified in 66.7% (34/51), most frequently impetigo (25.5%, 13/51) and nasal carriage (25.5%, 13/51). Trauma (17.7%, 9/51), parental nasal carriage (13.7%, 7/51) and less frequent entry points such as insect bites (5.9%, 3/51), omphalitis (2.0%, 1/51), erysipelas (2.0%, 1/51), and paronychia (2.0%, 1/51) were also documented.

### 3.4. Microbiological Findings and Antimicrobial Resistance

**Figure 2:**
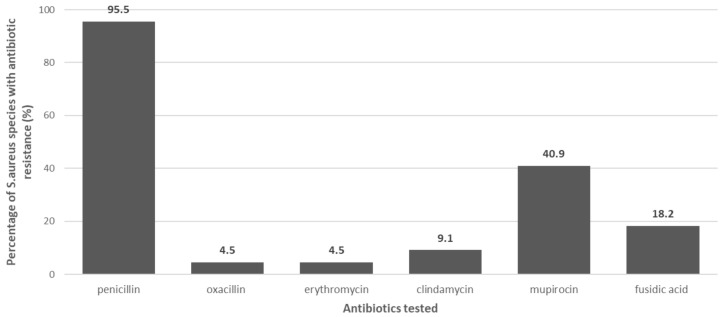
Antibiotic resistance rates (%) of *Staphylococcus aureus* isolates in hospitalized children with staphylococcal scalded skin syndrome in a tertiary pediatric hospital between 1/2009 and 12/2019.

*S. aureus* was isolated in 43.1% (22/51) of cases. No testing for exfoliative toxin was performed. All blood cultures were negative. Among the isolates tested, antibiotic resistance rates for *S. aureus* were as follows: penicillin 95.5% (21/22), oxacillin 4.5% (1/22), erythromycin 4.5% (1/22), clindamycin 9.1% (2/22), mupirocin 40.9% (9/22) and fusidic acid 18.2% (4/22) (Figure 2).

The antibiotic resistance of *S. aureus* isolates was analyzed over four time periods (2009–2011, 2012–2014, 2015–2017, and 2018–2019). Over time, mupirocin resistance increased gradually, from 0% in 2009–2011 and 2012–2014, to 11.1% in 2015–2017 and to 34.6% in 2018–2019 (*p* = 0.05). Clindamycin resistance also showed an upward trend, increasing from 0% in 2009–2011 to 11.5% in 2018–2019, although this was not statistically significant (*p* = 0.53). Resistance to oxacillin remained low and stable throughout the study period (*p* = 0.53). Resistance to fusidic acid and erythromycin likewise remained relatively low or stable over the years, with no significant changes observed. Using the non-penicillin resistance composite, prior antibiotic use was not associated with resistance [33.3% (5/15) with prior antibiotics vs. 27.8% (10/36) without prior antibiotics; *p* = 0.743].

Nasal carriage of *S. aureus* was detected in 34.0% (16/47) of children and 37.0% (10/27) of parents (Table 2). Parental *S. aureus* carriage was associated with child *S. aureus* carriage [60.0% (6/10) among children whose parents were carriers vs. 17.6% (3/17) among children whose parents were not carriers; OR 7.00, 95% CI 1.18–41.36; *p* = 0.039]. Child nasal carriage was also associated with microbiological documentation from cultures [62.5% (10/16) vs. 29.0% (9/31); OR 4.07, 95% CI 1.14–14.58; *p* = 0.034]. For resistance analyses, penicillin was excluded from the composite outcome. *S. aureus* resistant to at least one non-penicillin anti-staphylococcal agent was more frequent among child carriers than non-carriers [56.3% (9/16) vs. 12.9% (4/31); OR 8.68, 95% CI 2.05–36.69; *p* = 0.004].

### 3.5. Treatment

Intravenous clindamycin was the initial treatment in 58.8% (30/51). It was used as monotherapy in 49% (25/51) of cases, while combination therapy was used in 33.3% (17/51). At discharge, 35.3% (18/51) of children continued clindamycin. In 33.3% (17/51) of cases, nasal carriage was treated with a topical cream. Treatment modalities are shown in Table 3.

### 3.6. Complications and Outcome

In this study, complications occurred in 45.1% (23/51). Minor complications, including conjunctivitis, adverse drug reactions to antibiotics, and dehydration, were documented in all patients with complications (100%, 23/23), whereas major complications (hospital-acquired infections and ICU admission) occurred in 6 patients (6/23, 26.1%).

Mean (±SD) hospital stay was 6.3 ± 4.1 days, and treatment duration was 10.3 ± 2.2 days. Most cases had a positive outcome, with 98.0% (50/51) of children recovering successfully, while one child was transferred to the Neonatal Intensive Care Unit for further care. No deaths occurred.

### 3.7. Exploratory Association and Regression Analyses

Exploratory association and logistic regression analyses are summarized in Table 4. In a model adjusted for the late study period, child nasal *S. aureus* carriage remained associated with positive *S. aureus* culture (aOR 4.18, 95% CI 1.14–15.27; *p* = 0.031). In the subset with parental swabs, parental carriage was associated with child carriage, but was not independently associated with culture positivity after adjustment for child carriage (aOR 1.25, 95% CI 0.18–8.77; *p* = 0.822). Child nasal carriage and the 2018–2019 period were both associated with non-penicillin resistance in the exploratory model. For clinical outcomes, elevated inflammatory markers were independently associated with complications after adjustment for combination/advanced IV therapy (aOR 13.66, 95% CI 1.47–126.64; *p* = 0.021), whereas combination/advanced IV therapy itself was not independently associated with complications (aOR 2.39, 95% CI 0.70–8.15; *p* = 0.163). Children receiving combination/advanced IV therapy had longer hospitalizations than those not receiving such regimens [median 6.5 days (IQR 5.0–9.8) vs. 5.0 days (IQR 4.0–6.0); *p* = 0.016], most likely reflecting confounding by clinical severity.

## 4. Discussion

In the present study, we describe the epidemiological and microbiological characteristics of SSSS in children over an 11-year period in a tertiary hospital, enriching the limited available data on this rare condition.

The mean age of onset in our study was 2.5 years, aligning with literature indicating that SSSS predominantly affects children under five [6,7,8,10,11]. Proposed mechanisms for the higher incidence of SSSS in children include immature immunity (low anti-ET antibody titers), reduced renal toxin clearance, and skin barrier dysfunction, particularly in atopic dermatitis [2,3,12]. A slight female predominance was observed, though prior studies show variable sex distribution [3,6,10].

SSSS incidence showed a seasonal peak in summer and autumn, though this was not statistically significant. Similar patterns with seasonal variation in the US and China suggest possible environmental triggers [7,10]. More cases were recorded in non-urban areas, possibly reflecting disparities in healthcare access, socioeconomic factors, or environmental exposures [7,10].

A sharp increase in the incidence of SSSS was documented in 2018–2019. Similar trends have been reported internationally and may reflect the regional expansion of exfoliative toxin–producing *S. aureus* lineages. In Greece, the emergence of a toxinogenic methicillin-sensitive *S. aureus* (MSSA) clone (ST121) has previously been implicated in increased SSSS activity [9]. Comparable surges were observed in Texas, where SSSS cases rose more than eightfold between 2008 and 2014, driven predominantly by dissemination of ST121 strains, while North American and Belgian cohorts likewise reported rising rates of toxin-mediated *S. aureus* skin infections attributed to ET-producing clones [13,14,15]. In contrast, studies from Italy and Southwest China have reported stable or declining incidence, likely reflecting regional differences in circulating strains, diagnostic practices, and surveillance [6,16].

A personal history of atopic dermatitis was present in six patients; one had ichthyosis, and one developed nephrotic syndrome during hospitalization for SSSS. Atopic dermatitis is a recognized risk factor due to increased staphylococcal colonization, whereas only two neonatal and one adult cases of SSSS have been reported in association with ichthyosis and one pediatric case with nephrotic syndrome [17,18,19,20,21]. Despite renal disease, hospitalization lasted just five days, suggesting that prompt treatment can yield positive outcomes. Preceding upper respiratory tract infections were documented in over half of cases, consistent with prior studies [1,7,22]. Overall, a history of atopy, renal impairment, or recent infection may raise suspicion, though SSSS often occurs without clear risk factors [7,14,22].

*S. aureus* was isolated from 43.1% of cases in our study, consistent with studies from the US and Canada reporting yields between 40% and 74% [1,13,14,22]. Resistance to penicillin was high, reflecting widespread penicillinase production among *S. aureus* strains [10]. However, resistance to oxacillin, clindamycin and fusidic acid remained relatively low. Clindamycin was the most frequently prescribed antibiotic, chosen for its ability to achieve high skin concentrations and inhibit toxin production [19,22]. Monotherapy was mainly used in clinically stable patients with mild-to-moderate disease in a setting of low local MRSA prevalence, with close monitoring and prompt treatment escalation when clinically indicated [23]. Although its use as monotherapy remains controversial, recent studies suggest comparable efficacy and cost-effectiveness [5,22,24]. In our cohort, clindamycin resistance showed an upward trend, highlighting the need for ongoing surveillance and consideration of alternative or combination therapy in high-resistance settings [11]. Glycopeptides were reserved only for severe cases, treatment failure, or based on antimicrobial susceptibility results.

Mupirocin resistance rose, eliciting concern given its central role in decolonization strategies [5]. National and global data confirm increasing mupirocin resistance over the past decade, with a pooled prevalence of 7.6% among MSSA and 13.8% among MRSA, which may compromise eradication strategies [9,25,26,27,28]. Given that mupirocin remains the only approved agent for nasal decolonization of *S. aureus* and is also widely used as a topical treatment for mild skin infections, rising resistance poses risks for persistent colonization, recurrent infections, and household or healthcare-associated transmission [25,26,27]. From a practical standpoint, our findings support judicious mupirocin use, targeted rather than routine decolonization based on local susceptibility patterns whenever feasible, ongoing surveillance of antimicrobial resistance, and consideration of alternative or adjunctive decolonization strategies (e.g., chlorhexidine washes) in settings where mupirocin resistance is common.

The positive association between parental nasal carriage and child colonization aligns with previous reports that family members act as reservoirs for *S. aureus* transmission [29]. In the exploratory regression analysis, parental carriage was associated with child carriage but was not independently associated with positive *S.aureus* cultures after accounting for child carriage, suggesting that parental carriage may contribute indirectly through household transmission. Child nasal carriage, however, was associated with both microbiological confirmation and non-penicillin resistance. Penicillin was deliberately excluded from this composite because resistance was near universal and penicillin is no longer an informative marker of clinically relevant anti-staphylococcal resistance. These findings reinforce the key role of colonization in the epidemiology and pathogenesis of *S. aureus* infections, as carriage has been established as a major risk factor for infection, particularly among patients colonized with MRSA [30].

No deaths occurred in our cohort, supporting evidence that outcomes in children with SSSS are favorable with prompt treatment [4].

This study has several limitations. Its retrospective design introduced the possibility of selection and information bias, and reliance on discharge coding and documentation may have missed or misclassified mild or atypical cases, potentially underestimating the institutional incidence. Confirmation by a pediatric dermatologist likely reduced, but did not eliminate, diagnostic misclassification. Exfoliative toxin typing was unavailable. Cultures and parental nasal screening were not protocolized, and parental testing was performed in only 27 families. Because families with suspected household transmission may have been more likely to undergo screening, the observed parental carriage associations may be overestimated. Analyses used available cases without imputation, so non-random missingness may also have influenced the estimates. The incidence measure was standardized per hospitalization and therefore reflects institutional burden rather than population-based incidence. The regression analyses were exploratory and based on a limited cohort with sparse events, wide confidence intervals, and no adjustment for multiple comparisons; chance findings and model instability cannot be excluded. Treatment-related associations are additionally susceptible to confounding by indication and should not be interpreted as causal. Finally, the study covered the pre-COVID-19 period (2009–2019), and subsequent patterns may differ.

Accordingly, the findings are most generalizable to hospitalized children with SSSS managed in comparable tertiary pediatric centers and should not be extrapolated directly to community incidence, outpatient disease, or settings with different circulating *S. aureus* lineages and resistance patterns. Despite these limitations, the study has notable strengths, including a relatively large cohort for a rare condition, an extended 11-year observation period, complete in-hospital outcome data, and detailed microbiological and treatment information.

## 5. Conclusions

In conclusion, SSSS remains a rare but clinically significant condition in children. Our study identified a gradual increase in SSSS incidence that peaked in 2018–2019, rising mupirocin resistance, and higher clinically relevant non-penicillin antibiotic resistance among children with *S. aureus* nasal carriage, underscoring the potential role of colonization in transmission and antimicrobial resistance. Child nasal carriage was also associated with positive cultures from other sites, whereas elevated inflammatory markers were associated with complications. These exploratory associations should be regarded as hypothesis-generating rather than causal. Clindamycin monotherapy was commonly used, and overall outcomes were favorable in this setting; however, comparative effectiveness cannot be inferred from this observational design. Evolving resistance patterns emphasize the need for rational antibiotic use, regional surveillance data, and future multicenter studies with molecular analyses to clarify pathogenesis, resistance mechanisms, and optimal management.

## Figures and Tables

**Table 1:** Demographic and epidemiological characteristics of hospitalized children with staphylococcal scalded skin syndrome in a tertiary pediatric hospital between 1/2009 and 12/2019 (N = 51).

Characteristic	N (%)
Sex	
Male	25 (49.0)
Female	26 (51.0)
Age (years), mean (SD)	2.5 (2.0)
Age at Diagnosis	
<30 days	3 (5.9)
31 days–1 year	14 (27.5)
1–2 years	11 (21.6)
3–5 years	19 (37.3)
>5 years	4 (7.8)
Residence Type	
Rural	30 (58.8)
Urban	21 (41.2)

Notes: Data are presented as numbers (percentages) unless otherwise specified. SD = standard deviation. No data were missing for the variables presented in this table.

**Table 2:** Laboratory findings, patients’ and parents’ nasal carriage, and culture results among hospitalized children with staphylococcal scalded skin syndrome in a tertiary pediatric hospital between 1/2009 and 12/2019.

Category	N (%)
Laboratory Tests (N = 51)	
WBC < 15k/μL, CRP ≤ 20 mg/L	43 (84.3)
WBC > 15k/μL, CRP ≥ 20 mg/L	8 (15.7)
Patients’ Staphylococcal Carriage (N = 47)	
*Staphylococcus aureus*	16 (34.0)
*Staphylococcus epidermidis*	2 (4.2)
*Haemophilus influenzae*	1 (2.1)
*Moraxella catarrhalis*	1 (2.1)
*Proteus mirabilis*	1 (2.1)
*Pseudomonas aeruginosa*	1 (2.1)
Not isolated	25 (53.2)
Parents’ Staphylococcal Carriage (N = 27)	
None	16 (59.3)
*Staphylococcus aureus*	10 (37.0)
Other	1 (3.7)
Not tested	24

Notes: WBC = white blood cell count; CRP = C-reactive protein. Data are presented as number (percentage) unless otherwise indicated. Child nasal carriage data were unavailable for 4 participants, and parental screening data were unavailable for 24 families; percentages are based on the available denominators.

**Table 3:** Treatment modalities in hospitalized children with staphylococcal scalded skin syndrome in a tertiary pediatric hospital between 1/2009 and 12/2019.

Category	N (%)
Antibiotic Therapy (N = 51)	
IV Clindamycin	27 (52.9)
IV Clindamycin + Cloxacillin	3 (5.9)
IV Clindamycin + Cefotaxime	4 (7.8)
IV Clindamycin + Teicoplanin	9 (17.6)
IV Clindamycin with switch to Teicoplanin	3 (5.9)
IV Glycopeptide (Teicoplanin or Vancomycin)	3 (5.9)
PO Fusidic Acid	1 (2.0)
PO Penicillin	1 (2.0)
Therapeutic Approach (N = 51)	
Monotherapy	25 (49.0)
Dual therapy	17 (33.3)
Dual therapy initially, then monotherapy	3 (5.9)
Switch due to resistance	0 (0.0)
Switch due to lack of response	2 (3.9)
Initial monotherapy, adjusted by antibiogram	4 (7.8)
Type of Discharge Treatment (N = 51)	
No discharge treatment	6 (11.8)
Clindamycin	18 (35.3)
Amoxicillin/Clavulanic Acid	16 (31.4)
Trimethoprim	1 (2.0)
Clarithromycin	1 (2.0)
Fusidic Acid	2 (3.9)
Cefuroxime	5 (9.8)
Clindamycin & Cefuroxime	1 (2.0)
Clindamycin & Rifampicin	1 (2.0)
Topical Treatment of Carriage (N = 51)	
No treatment	34 (66.7)
Mupirocin	16 (31.4)
Fusidic Acid	1 (2.0)

Notes: IV = intravenous; PO = per os (oral).

**Table 4:** Exploratory association and logistic regression analyses for microbiological confirmation, non-penicillin resistance, and complications.

Dependent Variable	Independent Variable	Event Rate: Exposed vs. Unexposed	Unadjusted OR (95% CI); *p*	Adjusted OR (95% CI); *p*
Positive *S. aureus* culture	Child nasal *S. aureus* carriage	10/16 (62.5%) vs. 9/31 (29.0%)	4.07 (1.14–14.58); *p* = 0.034	4.18 (1.14–15.27); *p* = 0.031, adjusted for 2018–2019
Positive *S. aureus* culture	Parental nasal *S. aureus* carriage	6/10 (60.0%) vs. 6/17 (35.3%)	2.75 (0.55–13.75); *p* = 0.257	1.25 (0.18–8.77); *p* = 0.822, adjusted for child carriage
Child nasal *S. aureus* carriage	Parental nasal *S. aureus* carriage	6/10 (60.0%) vs. 3/17 (17.6%)	7.00 (1.18–41.36); *p* = 0.039	Not modeled
Non-penicillin resistance	Child nasal *S. aureus* carriage	9/16 (56.3%) vs. 4/31 (12.9%)	8.68 (2.05–36.69); *p* = 0.004	25.68 (2.64–249.50); *p* = 0.005, adjusted for 2018–2019
Non-penicillin resistance	2018–2019 period	13/26 (50.0%) vs. 2/25 (8.0%)	11.50 (2.24–59.09); *p* = 0.002	29.81 (2.63–338.22); *p* = 0.006, adjusted for child carriage, N = 47
Any complication	Elevated WBC/CRP	7/8 (87.5%) vs. 16/43 (37.2%)	11.81 (1.33–104.99); *p* = 0.016	13.66 (1.47–126.64); *p* = 0.021, adjusted for combination/advanced IV therapy
Any complication	Combination/advanced IV therapy	12/22 (54.5%) vs. 11/29 (37.9%)	1.96 (0.64–6.05); *p* = 0.269	2.39 (0.70–8.15); *p* = 0.163, adjusted for elevated WBC/CRP

Notes: Penicillin was excluded from the composite resistance outcome. Non-penicillin resistance was defined as resistance to at least one of oxacillin, erythromycin, clindamycin, mupirocin, or fusidic acid. Adjusted models were parsimonious because of the small sample size and sparse events. OR = odds ratio; CI = confidence interval; WBC = white blood cell count; CRP = C-reactive protein; IV = intravenous. All association and regression analyses used available cases; the analytic denominator therefore varied according to the availability of child and parental carriage data.

## Data Availability

The datasets used and analyzed during the current study are available from the corresponding author on reasonable request.

## Supplementary Materials

The following supporting information can be downloaded at: https://www.mdpi.com/article/10.3390/children13070965/s1, File S1: Completed STROBE Checklist for Cohort Studies.

## Abbreviations

The following abbreviations are used in this manuscript:

SSSS	Staphylococcal scalded skin syndrome
*S. aureus*	*Staphylococcus aureus*
MRSA	Methicillin-resistant *Staphylococcus aureus*
ET	Exfoliative Toxin
ET-A	Exfoliative toxin A
ET-B	Exfoliative toxin B
NICU	Neonatal Intensive Care Unit
ICD-10	International Classification of Diseases, 10th Revision
SD	Standard Deviation
IQR	Interquartile Range
SPSS	Statistical Package for the Social Sciences
CLSI	Clinical and Laboratory Standards Institute
PBP2a	Penicillin-Binding Protein 2a
US	United States
STROBE	Strengthening the Reporting of Observational Studies in Epidemiology
