# Supplementary material for: Staphylococcal Scalded Skin Syndrome in a Tertiary Pediatric Hospital over an 11-Year Period
Source: Children (Basel). 2026 Jul 21;13(7):965. doi: 10.3390/children13070965 (PMC13406476; doi:10.3390/children13070965)
Supplement: Supplementary file 1 [file children-13-00965-s001.zip › children-4407769-supplementary.pdf]

Completed STROBE Checklist for Cohort Studies

Staphylococcal Scalded Skin Syndrome in a Tertiary Pediatric Hospital Over an 11-Year Period

Item	Reporting requirement	Manuscript location	How addressed
1(a)	Identify the observational design in the title or abstract.	Abstract, pp. 1–2	Reported as a retrospective cohort study in the Abstract.
1(b)	Provide a balanced summary of methods and findings.	Abstract, pp. 1–2	Design, participants, analyses, principal results, and conclusions are summarized.
2	Explain the scientific background and rationale.	Introduction, pp. 2–3	Epidemiology, pathophysiology, diagnosis, treatment, and the Greek evidence gap are described.
3	State the study objectives and any prespecified hypotheses.	Introduction, p. 3	Objectives include epidemiology, microbiology, resistance, carriage, treatment, and complications; exploratory associations are specified.
4	Present key elements of the study design early.	Methods, Section 2.1, pp. 3–4	Retrospective, single-center observational cohort design is stated at the start of Methods.
5	Describe setting, locations, and relevant dates.	Methods, Section 2.1, pp. 3–4	Hospital, bed capacity, admission dates, and admission-to-discharge observation period are reported.
6(a)	Give eligibility criteria and participant-selection methods; describe follow-up.	Methods, Section 2.2, pp. 3–4	ICD-10 identification, age range, clinical criteria, dermatologist confirmation, inclusion rules, and in-hospital observation are described.
6(b)	For matched studies, report matching criteria and group numbers.	*Not applicable*	The study was not matched.
7	Define outcomes, exposures, predictors, confounders, and diagnostic criteria.	Methods, Section 2.4, p. 4	Incidence, culture confirmation, carriage, resistance, inflammatory markers, complications, treatment intensity, and diagnostic criteria are defined.
8	Describe data sources and measurement methods for each variable.	Methods, Sections 2.3 and 2.5, pp. 4–5	Medical records, administrative denominators, sampling sites, culture methods, and susceptibility procedures are specified.
9	Describe efforts to address potential sources of bias.	Methods, Section 2.6, p. 5	Diagnostic confirmation, inclusion of all eligible admissions, clinician-directed testing, and ascertainment bias are addressed.
10	Explain how the study size was determined.	Methods, Section 2.6, p. 5	The cohort was a census of all eligible SSSS admissions; no a priori calculation was performed.
11	Explain handling and categorization of quantitative variables.	Methods, Sections 2.4 and 2.7, pp. 4–6	Continuous summaries, pediatric age groups, inflammatory-marker thresholds, and calendar periods are described.
12(a)	Describe statistical methods, including control of confounding.	Methods, Section 2.7, pp. 5–6	Tests, logistic regression, predictor limits, adjustment variables, ORs/aORs, and confidence intervals are reported.
12(b)	Describe subgroup and interaction analyses.	Methods, Section 2.7; Results, Sections 3.2 and 3.4	Temporal subgroup analyses are described. No interaction analyses were performed.
12(c)	Explain how missing data were handled.	Methods, Section 2.7, p. 6; Results, Section 3.1 and table notes	No imputation; available-case denominators are reported for child and parental carriage.
12(d)	Explain handling of loss to follow-up.	Methods, Sections 2.1 and 2.7, pp. 3 and 6	Outcomes were assessed during hospitalization; outcome data were complete and loss to follow-up was not applicable.
12(e)	Describe sensitivity analyses.	Methods, Section 2.7, p. 6	No formal sensitivity analyses were performed because of the limited, exploratory dataset.
13(a)	Report participant numbers at each study stage.	Results, Section 3.1, p. 6	The final cohort of 51 eligible children and testing denominators are reported.
13(b)	Give reasons for non-participation at each stage.	Methods, Section 2.2, pp. 3–4	All records meeting the prespecified criteria were included; no additional non-participation stage occurred.
13(c)	Consider a participant flow diagram.	Results, Section 3.1, p. 6	Flow is reported narratively because case ascertainment was single-stage and there was no follow-up attrition.
14(a)	Report participant characteristics and relevant exposures/confounders.	Results, Sections 3.1–3.6; Tables 1–3, pp. 6–10	Demographic, clinical, laboratory, microbiological, treatment, and outcome characteristics are provided.

Item	Reporting requirement	Manuscript location	How addressed
14(b)	Report numbers with missing data for variables of interest.	Results, Section 3.1; notes to Tables 1, 2, and 4	Missing child and parental carriage data and available denominators are stated.
14(c)	Summarize follow-up time.	Methods, Section 2.1; Results, Section 3.6	Observation was from admission to discharge; hospital-stay duration is reported.
15	Report outcome-event numbers or summary measures over time.	Results, Sections 3.2–3.6, pp. 6–10	Annual incidence, resistance, complications, treatment, length of stay, recovery, ICU transfer, and mortality are reported.
16(a)	Give unadjusted and adjusted estimates with precision and identify confounders.	Results, Section 3.7 and Table 4, pp. 10–12	Unadjusted ORs and adjusted ORs with 95% CIs and adjustment variables are reported.
16(b)	Report boundaries when continuous variables are categorized.	Methods, Section 2.4; Tables 1–2	Age categories and WBC/CRP thresholds are stated.
16(c)	When relevant, translate relative estimates into absolute risk.	*Not applicable*	The analyses report odds ratios for exploratory associations; an absolute-risk translation was not applicable.
17	Report other analyses, including subgroup or sensitivity analyses.	Results, Sections 3.2, 3.4, and 3.7, pp. 6–12	Temporal, resistance-period, association, and regression analyses are reported; no sensitivity analyses were conducted.
18	Summarize key results with reference to the objectives.	Discussion and Conclusions, pp. 12–14	Key epidemiological, microbiological, resistance, carriage, and complication findings are summarized.
19	Discuss limitations, including direction and magnitude of potential bias or imprecision.	Discussion, pp. 13–14	Selection, information, ascertainment and missing-data bias, sparse events, wide CIs, multiplicity, and confounding by indication are discussed.
20	Provide a cautious interpretation considering objectives, limitations, multiplicity, and other evidence.	Discussion and Conclusions, pp. 12–14	Associations are described as exploratory and hypothesis-generating; causal and comparative-effectiveness claims are avoided.
21	Discuss generalisability or external validity.	Discussion, p. 14	Applicability to comparable tertiary pediatric centers and limits for community/outpatient settings are stated.
22	Report funding source and the role of funders.	Funding statement, p. 14	No external funding; no funder role in any study or publication stage.
